# Application of through-the-scope twin clip for semicircumferential defect closure after rectal laterally spreading tumor transanal super minimally invasive surgery resection

**DOI:** 10.1055/a-2443-4236

**Published:** 2024-11-08

**Authors:** Chenyang Li, Ningli Chai, Tao Wang, Shuling Li, Fuxiu Huang, Ningning Zhang, Chao Chen

**Affiliations:** 1 Department of Gastroenterology, First Medical Center of PLA General Hospital, Beijing, China; 2 Department of Gastroenterology, Fourth Medical Center of PLA General Hospital, Beijing, China

With increasing defect depth and size, the complexity of closure intensifies. While the over-the-scope clip is a favored approach for larger defects, it encounters challenges such as pre-procedure attachment. This requirement can prolong surgical time and raise the risk of postoperative complications, including peritonitis 1 .

We report the application of the through-the-scope twin clip (TTS-TC) for semicircumferential defect closure after dissection of a laterally spreading tumor (LST). The device was initially developed and implemented by Prof. Zhang’s team 2 . The device features a central support column that enables independent operation of its bilateral clips, with a maximum outer diameter of 2.9 mm, an opening angle of up to 60 degrees, and an opening size of 1.0 cm, making it suitable for effective closure of defects less than 5.0 cm in diameter 3 4 5 .

A 65-year-old woman was admitted to our hospital with a 3.5 × 3.1 cm LST in the rectum. We resected the lesion using super minimally invasive surgery (SMIS), also known as endoscopic submucosal dissection. To address the post-SMIS mucosal defect, which measured 4.5 × 4.5 cm and extended over half the circumference, we elected to utilize TTS-TCs as a means of effectively closing the gap ( Fig. 1 , Video 1 ).

**Fig. 1:**
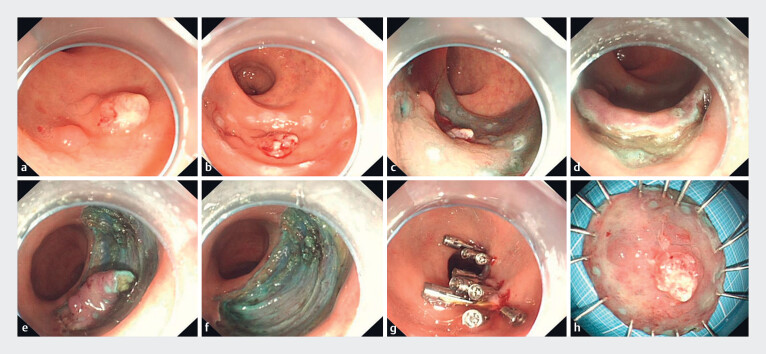
Procedure steps for transanal super minimally invasive surgery resection of a rectal laterally spreading lesion (LST). **a** An LST was located in the rectum. **b** Marking the mucosa surrounding the LST. **c** Injecting fluid into the submucosa. **d** Mucosal incision was performed. **e** The submucosa was dissected. **f** A semicircumferential defect was formed. **g** Closure of the defect using through-the-scope twin clip and traditional clips. **h** The LST specimen.

Closure of a semicircumferential defect with the novel through-the-scope twin clip after super minimally invasive surgery in the rectum.Video 1

We inserted the TTS-TC through the endoscope channel and clamped it to one side of the defect, positioned the other side, and then released the TTS-TC. The large defect then transformed into smaller defects. Subsequently, several traditional through-the-scope clips were deployed to completely close the defect.

The patient fasted for 1 day and was discharged after 3 days without any postoperative complications. The histological examination disclosed a colonic adenoma exhibiting high grade intraepithelial neoplasia, with localized regions of invasive carcinoma.

The TTS-TC emerges as a promising tool for the closure of large colorectal defects, offering a new dimension in endoscopic management.

Endoscopy_UCTN_Code_TTT_1AQ_2AK

## Notes

Endoscopy E-Videos https://eref.thieme.de/e-videos
*E-Videos* is an open access online section of the journal *Endoscopy* , reporting on interesting cases and new techniques in gastroenterological endoscopy. All papers include a high-quality video and are published with a Creative Commons CC-BY license. Endoscopy E-Videos qualify for HINARI discounts and waivers and eligibility is automatically checked during the submission process. We grant 100% waivers to articles whose corresponding authors are based in Group A countries and 50% waivers to those who are based in Group B countries as classified by Research4Life (see: https://www.research4life.org/access/eligibility/ ). This section has its own submission website at https://mc.manuscriptcentral.com/e-videos .
